# The Dysfunction of CD4^+^CD25^+^ Regulatory T Cells Contributes to the Abortion of Mice Caused by *Toxoplasma gondii* Excreted-Secreted Antigens in Early Pregnancy

**DOI:** 10.1371/journal.pone.0069012

**Published:** 2013-07-17

**Authors:** Jin-ling Chen, Yi-yue Ge, Jie Zhang, Xiao-yan Qiu, Jing-fan Qiu, Jiang-ping Wu, Yong Wang

**Affiliations:** 1 Department of Pathogen Biology, Key Laboratory of Pathogen Biology of Jiangsu Province, Nanjing Medical University, Nanjing, Jiangsu, China; 2 Department of Parasitology and Microbiology, School of Medicine, Nantong University, Nantong, Jiangsu, China; 3 Institute of Pathogen Microbiology, Jiangsu Provincial Center for Disease Prevention and Control, Nanjing, Jiangsu, China; 4 Nanjing Maternity and Child Health Care Hospital Affiliated to Nanjing Medical University, Nanjing, Jiangsu, China; University of Kentucky, United States of America

## Abstract

*Toxoplasma gondii* is an opportunistic intracellular parasite that is highly prevalent in human and warm-blooded animals throughout the world, leading to potentially severe congenital infections. Although the abortion caused by *T. gondii* is believed to be dependent on the timing of maternal infection during pregnancy, the mechanism remains unclear. This study was focused on the effects of *T. gondii* excreted-secreted antigens on pregnant outcomes and CD4^+^CD25^+^ Foxp3^+^ regulatory T cells at different stages of pregnancy. The results showed that in mice the frequency and suppressive function of CD4^+^CD25^+^ regulatory cells were diminished after injection of *T. gondii* excreted-secreted antigens at early and intermediate stages of pregnancy. The abortion caused by *T. gondii* excreted-secreted antigens at early pregnancy could be partly prevented by adoptively transferring of CD4^+^CD25^+^ cells from the mice injected with *T. gondii* excreted-secreted antigens at late pregnancy, but not from the mice with the same treatment at early pregnancy. Furthermore, *T. gondii* excreted-secreted antigens induced apoptosis of CD4^+^CD25^+^ regulatory cells of mice in early and intermediate stages of pregnancy by down-regulating their Bcl-2 expressions and Bcl-2/Bax ratio. This study provides new insights into the mechanism that *T. gondii* infection is the high risk factor for abortion in early pregnancy.

## Introduction

*Toxoplasma gondii* (*T. gondii*) is an obligate intracellular protozoal parasite. Infection with *T. gondii* can lead to severe disease, such as pneumonia and encephalitis, in immunocompromised hosts [1]. *T. gondii* infection may cause maternal immune deregulation and a variety of syndromes during pregnancy, such as miscarriage, spontaneous abortion, or fetal teratogenesis [2]. Moreover, the severity of congenital toxoplasmosis depends on the stage of pregnancy at which infection takes place [3]. Importantly, this phenomenon is not limited to *T. gondii* infection. The impact of other infectious agents in the TORCH group on the pathogenesis of such event is well known [4], [5]. Although previous reports have indicated that the abortion is closely relevant to the timing of maternal infection during pregnancy, the molecular mechanism remains unclear.

During gestation, the maternal immune system normally tolerates the paternal alloantigens. Several specialized mechanisms, such as depleting tryptophan [6], inactivating NK cells through HLA-G expression [7], or provoking apoptosis of activated maternal lymphocytes [8] were proposed as having contributed to such a tolerance. Tafuri et al. reported that the maternal immune system could specifically tolerate the engraftment of paternally- derived tumor cells and reject the tumor grafts after delivery [9], suggesting that the tolerance specific to paternal alloantigens is restricted to the pregnancy period. Thus, in addition to locally acting mechanisms, systemic maternal immune system must be altered to facilitate fetal tolerance [9], [10].

CD4^+^CD25^+^ regulatory T cells (Tregs) were claimed to be important players in the tolerance towards the fetus bearing alloantigens [11], [12], [13]. Diminished number of Tregs was associated with immunological rejection of fetus, which could be prevented by adoptively transferring Tregs from normal pregnant mice into abortion-prone animals [14], [15]. Our previous study demonstrated that CD4^+^CD25^+^ T cells were involved in the pathogenesis of abortion caused by *T. gondii*. Foxp3 gene, as a master regulator of Tregs, its expression levels decreased in splenocytes and placentas of the infected mice. We wonder if CD4^+^CD25^+^ T cells have contributed to the mechanism that the abortion caused by *T. gondii* is closely dependent on the timing of maternal infection during pregnancy. Importantly, a line of studies reveals that early fetal resorption is not due to a direct effect of uterine *T. gondii* proliferation, but other mechanisms [16], [17].Thus, in our study, in order to rule out the possibility that the abortion was caused by vertical infection, *T. gondii* ESA, which constitutes mostly of the circulating antigens in acutely infected hosts, and thus one of the first targets of the immune response [18], [19], was injected into mice at different pregnant stages. We sought to determine whether *T. gondii* ESA injection at different pregnant stages can differently influence CD4^+^CD25^+^ regulatory T cells and then lead to different pregnancy outcomes.

## Methods

### Mice and Mating

Female 6-8-week old and male 8-10-week old C57BL/6 mice were purchased from the Centre of Experimental Animals, Yangzhou University (Yangzhou, China). Mice were bred with free access to water and food under conditions of controlled temperature (22°C±2°C) and humidity (50%±10%), under a 12∶12-hour light-dark cycle, in the Laboratory Animal Center at Nanjing Medical University. All animal experiments were approved by the Institutional Animal Experimental Ethics Committee of Nanjing Medical University (N2011503). After 1 week of acclimation, female and male mice were paired in the evening. In the next morning, confirmation of a vaginal plug was defined as day 0 of pregnancy. Normal and absorbed implantation sites were identified by visual observation. An implantation site with a shrunk placenta and a dissolved or discolored brown embryo was defined as an abortion site [20]. The number of both types of sites was counted on gestational day 18. The percentage of abortions was calculated as the ratio of resorption sites to the total number of implantation sites (resorption plus normal implantation sites) as described previously [21], [22].

### *T. gondii* and ESA Preparation

*T. gondii* RH strain tachyzoites were maintained in mice by intraperitoneal inoculation every 3 days [23]. *T. gondii* ESA was prepared according to GE et al [17]. The *T. gondii* ESA was treated by AffinityPak Detoxi-Gel Endotoxin Removing Gel (Thermo, fairlawn, OH, USA) to remove endotoxin. The endotoxin of *T. gondii* ESA was 0.01 EU/kg, and lower than 0.2 EU/kg according to the endotoxin normative standard in ‘American FDA finally product examination guide’ [24]. Then the ESA was dissolved in PBS. The protein concentration of ESA was 0.933 mg/ml, as determined by bicinchoninic acid protein assay (Pierce, Rockford, IL). The same batch of ESA prepared was used throughout the study. A total of 0.1 ml of ESA was injected intraperitoneally (ip) into pregnant mice at gestational day 5 (G5), day 10 (G10) and day 15 (G15), respectively. The injection of same volume of PBS was as control.

### Flow Cytometric Analysis

After the injection of *T. gondii* ESA or PBS at G5, G10 and G15, respectively, mice were sacrificed at G18. Spleens, inguinal lymph nodes and peripheral blood from the mice were collected, and single-cell suspensions were prepared according to Tang et al [25]. For the analysis of CD4^+^CD25^+^Foxp3^+^ T-cell, the Mouse Regulatory T Cell Staining Kit was used following the instructions of the manufacturer (eBioscience, San Diego, CA, USA). For the analysis of apoptosis, cells (10^6^) were stained with anti-CD4–PE and anti-CD25–APC, respectively, washed, and then stained with FITC-labeled Annexin V and 7AAD (eBioscience). CD4^+^CD25^+^ events were collected for annexin/7AAD analysis. CD4^+^CD25^+^ cells in early apoptosis (annexin^+^7AAD^−^) were in the lower right quadrant. Live cells (annexin^−^7AAD^−^) were in the lower left quadrant. Dead cells (annexin^+^7AAD^+^) were in the upper right quadrant.

### Isolation of Tregs and Adoptive Transfer Experiment

CD4^+^CD25^+^ T cells were isolated from splenocytes of normal pregnant or abortion-prone mice by using magnetic beads following the manufacturer’s instructions (MACS, Miltenyi Biotech, Germany). The purity of the preparations was between 96% and 98% in all experiments. The percentage of CD4^+^CD25^+^Foxp3^+^ in CD4^+^CD25^+^ T cells was 82%. After isolation, pregnant mice injected with *T. gondii* ESA at G5 were transferred intravenously with 2×10^5^ CD4^+^CD25^+^ T cells in 200 µl of PBS. The pregnancy outcomes were observed at G18.

### Real-time Quantitative PCR

For real-time quantitative PCR analysis, total RNA was isolated from placentas using Trizol reagent (Invitrogen, San Diego, CA), and cDNA was synthesized with Moloney murine leukaemia virus reverse transcriptase and an oligo-d (T)15 primer (Promega, Madison, WI). A target cDNA sample was added to SYBR Green PCR master mix (Applied Biosystems, Foster city, CA) to generate quantitative gene expression data on an ABI Prism 7300 sequence detection system (Applied Biosystems). An amplification reaction was performed in a total volume of 20 µL for 40 cycles. All samples were run in triplicate and the relative expression levels were determined by normalization to β-actin and presented as fold increase or decrease relative to the controls. Primer sequences used were as follows: β-actin, forward: GCTCTGGCTCCT AGCACCAT; reverse: GATCCACACAGAGTACTTGCGC. Foxp3, forward: GGCCCTTCTCCAGGACAGA; reverse: GCTGAT CATGGCTGGGTTGT [26]. Caspase 3, forward: TCTGACTGGAAAGCCGAAACT; reverse: AGGGAC TGGATGAACCACGAC [27].

### Western Blot Analysis

CD4^+^CD25^+^ T cells and placentas were washed in PBS, then lysed in lysis buffer (25 mM Tris, pH 8.5, 2% lithium dodecyl sulfate, 1 mM EDTA, 10 mM sodium fluoride, 1 mM sodium orthovanadate, and 1×complete protease inhibitors) and quantified by bicinchoninic acid protein assay (Pierce, Rockford, IL). Lysates were separated on 4–15% SDS–polyacrylamide gel electrophoresis (PAGE) gels and transferred to PVDF (IPVH00010, Millipore, USA) followed by blocking in TBS/0.1% Tween 20 with 5% non-fat dry milk. Rabbit anti-mouse Foxp3 antibody (1∶2000) (Abcam, Cambridge, MA), Bax antibody (1∶1000), Bcl-2 antibody (1∶1000), Caspase 3 antibody(1∶1000) and goat anti-rabbit IgG HRP-conjugated antibody (1∶3000) (all manufactured by Cell Signaling Technology) were used for the detection of proteins. Glyceraldehyde-3- phosphate dehydrogenase (GAPDH) or β-actin was detected with mouse anti-GAPDH antibody (1∶1000) or anti-β-actin antibody (1∶5000) (both manufactured by Epitomics) as an internal control.

### Immunohistochemistry

Immediately following euthanasia of pregnant mice, placentas were fixed in 4% (w/v) buffered formaldehyde, embedded in paraffin. The sections were deparaffinized with xylene rehydrated, and transferred to water. Samples were preheated in citrate buffer (10 mmol/L, pH 6.0) for antigen retrieval and then treated with Protein Blocking Agent to reduce the nonspecific binding of antibodies. After washing, sections were incubated with purified anti-Foxp3 antibody (Abcam, Cambridge, MA) for 1 h, washed in PBS, and incubated with an Alkaline Phosphatase -labeled Goat Anti-Rabbit IgG as secondary antibody (Beyotime Institute of Biotechnology, Jiangsu, China). Visualization of the antigen–antibody complex was achieved by incubating sections in BCIP/NBT Alkaline Phosphatase Color Development Kit (Beyotime Institute of Biotechnology) for 10 - 30 min, until a satisfactory colour developed. All steps were performed at room temperature. Negative controls were samples in which the primary antibody was replaced with 10% bovine serum albumin. All sections were analyzed under light microscopy by two observers. The average number of Foxp3^+^ cells in four random high-power fields was determined for each sample.

### *In vitro* Proliferation Assay

Mitomycin C-treated, T-depleted splenocytes (2×10^5^) were used as APCs. CD4^+^CD25^−^ T cells (1×10^5^) purified from control mice were stimulated with 1 µg/ml anti-CD3 mAb (145 2c11, BD Pharmingen) in the presence of CD4^+^CD25^+^ T cells (1×10^5^) isolated from control or ESA-injected pregnant mice and cultured for 72 h. Cultures were performed in round-bottom 96 well plate (Corning, Costar) in complete medium: RPMI1640 (Gibco BRL, Gaithersburg, USA) supplemented with 10% heat-inactivated FCS (Sigma, St Louis, USA) and 100 U/ml penicillin-streptomycin. 1 µCi of [^3^H] TdR (Amersham Bioscience) was added to the well in the last 16 h of culture. [^3^H]TdR incorporation was measured with a Matrix 96 Direct Beta Counter (Packard).

### Cytokine Assays

At the indicated time, blood was collected from pregnant mice injected with *T. gondii* ESA. Serum levels of IFN-γ and IL-4 were measured by using a Mouse Th1/Th2 ELISA Ready-SET-Go kit according to the manufacturer’s instructions (eBioscience).

### Statistical Analysis

Prism software (GraphPad) was used to determine the statistical significance of differences in the means of experimental groups. Differences in abortion rates were determined by using the nonparametric Mann–Whitney U-test. Data of two groups were analyzed for statistical significance with Student’s t-test. Multiple comparisons were made by using one-way ANOVA.

## Results

### Injection of *T. gondii* ESA at Different Stages of Pregnancy Leads to Different Pregnancy Outcomes of Mice

**Figure 1:**
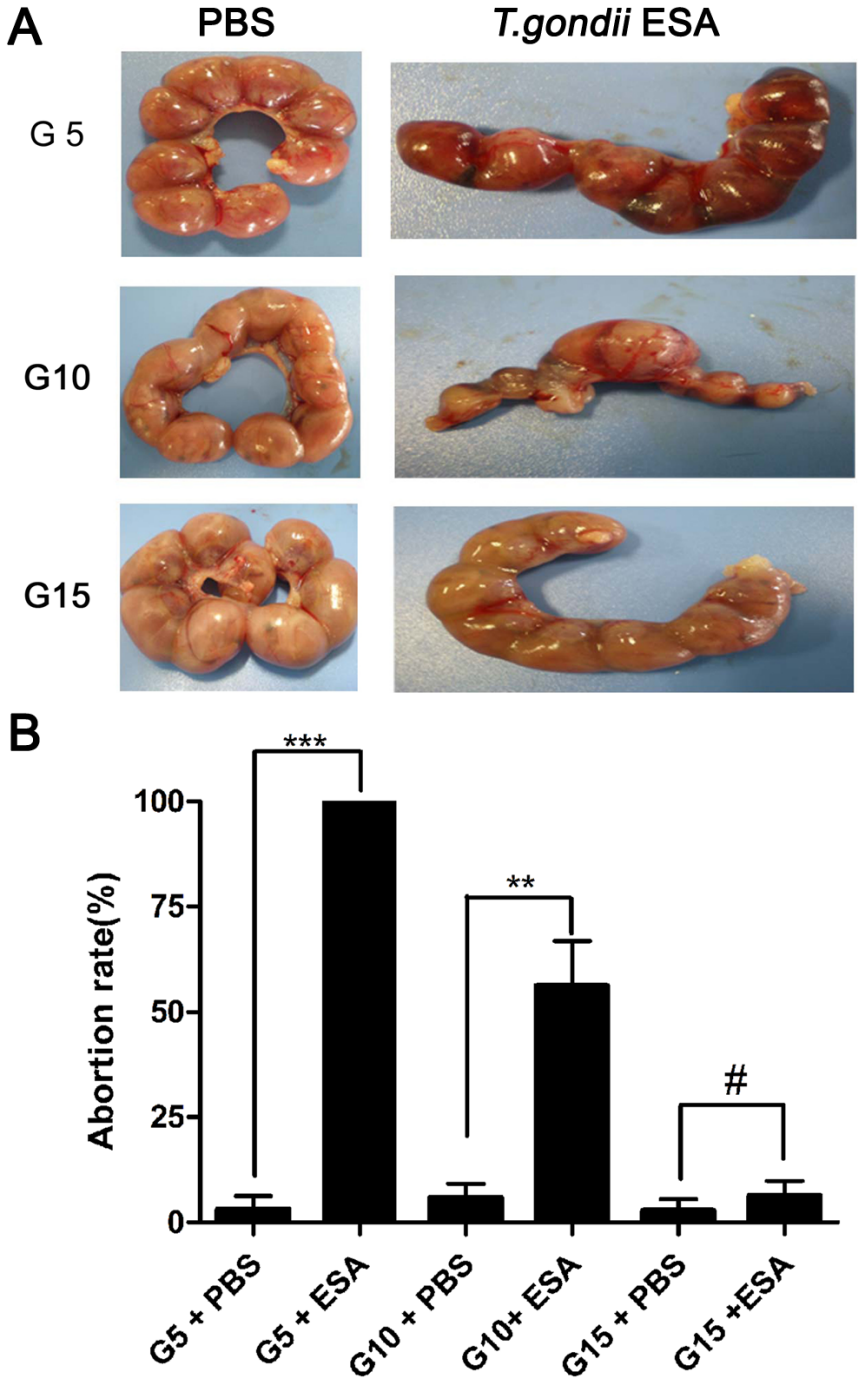
The abortion rate of pregnant mice after *T. gondii* ESA injection. (A) Representative pictures of uteri from mice with *T. gondii* ESA or PBS injection at gestational day 5(G5), day 10(G10) and day 15(G15), respectively. All the animals were sacrificed at G18. (B)The abortion rates calculated as the ratio of abortion sites to the total numbers of implantation sites after the injection of *T. gondii* ESA at G5, G10, and G15. Statistical differences between groups are shown as follows: ** *p*<0.01; *** *p*<0.001; ^#^ *p*>0.05.

*T. gondii* ESA or PBS was injected intraperitoneally (ip) into pregnant mice at G5, G10 and G15, respectively. The animals were sacrificed at G18. All fetuses and placentas became necrotic and haemorrhagic, and were resorbed after the administration of *T. gondii* ESA intraperitoneally at G5 (G5 ip), with the abortion rate of nearly 100%. Some embryos and placentas exhibited a necrotic and haemorrhagic appearance after the administration of *T. gondii* ESA intraperitoneally at G10 (G10 ip), with the abortion rate up to 56.20%. However, after the injection of *T. gondii* ESA at G15, there was no visible fetal abnormality in pregnant mice, which was consistent with the mice in control group (Figure 1).

### Injection of *T. gondii* ESA at the Early and Intermediate Stages of Pregnancy Reduces the Frequency and Function of CD4^+^CD25^+^Foxp3^+^ T Cells of Mice

**Figure 2:**
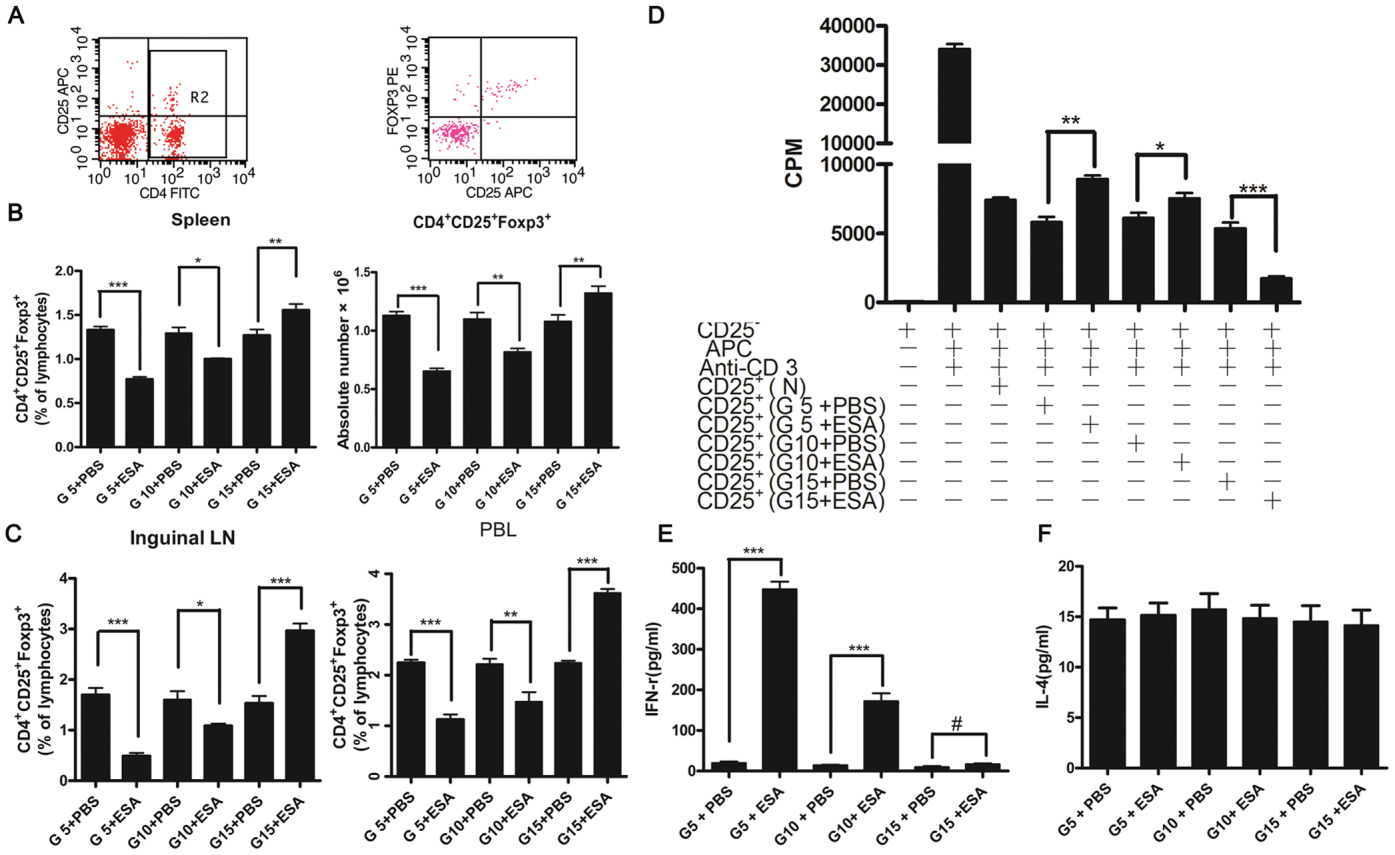
Effects of *T. gondii* ESA on the proportion and function of CD4^+^CD25^+^Foxp3^+^ T cells at different stages of pregnancy. All animals were killed at G18, and their spleens, inguinal LN, and PBL were obtained. Lymphocytes from these tissues were prepared and pooled as described in Materials and Methods. The cells were stained with CD4-FITC, CD25-APC and PE-Foxp3 Abs, respectively, and analyzed by flow cytometry. (A)Representative dot plots illustrating the regions and gating for the capture of cell phenotype data and intracellular Foxp3 expression. (B) Percentages and absolute number of CD4^+^CD25^+^Foxp3^+^-cells from spleens. (C) Percentages of CD4^+^CD25^+^Foxp3^+^-cells from inguinal LN, and PBL. (D) Responder CD4^+^CD25^–^ T cells (1×10^5^/well) from naive mice were cultured with naive, irradiated APC (1×10^5^ cells/well) and CD4^+^CD25^+^T cells (5×10^4^ cells/well) harvested from mice with PBS or *T. gondii* ESA injection at G5, G10, G15, respectively. (E and F) The serum levels of IFN-γ and IL-4 in pregnant mice injected with *T. gondii* ESA by ELISA. Data represent means ± SD from groups of seven mice assayed individually. Statistical differences between groups are shown as follows: * *p*<0.05; ** *p*<0.01; *** *p*<0.001; ^#^ *p*>0.05.

It has been previously determined that *T. gondii* has the ability to diminish the number of CD4^+^CD25^+^Foxp3^+^ T cells of mice during the gestation [17]. Consistent with those data, we found that the administration of *T. gondii* ESA at early (G5) and intermediate (G10) stages of pregnancy could also lead to the decrease of CD4^+^CD25^+^Foxp3^+^ T cells. However, after the injection of *T. gondii* ESA at the late pregnancy (G15), the percentage of CD4^+^CD25^+^Foxp3^+^ T cells increased compared with that of the control group (Figure 2A). The phenomenon could also be observed in the inguinal lymph nodes (LN) and peripheral blood lymphocytes (PBL) (Figure 2B and 2C), suggesting that *T. gondii* ESA induced global changes of CD4^+^CD25^+^Foxp3^+^ T cells. Next, we tested whether the regulatory function of these cells from the injected group of mice had been damaged by evaluating the suppressing proliferation of CD4^+^CD25^+^ T cells *in vitro* and Th2/Th1-like responses *in vivo*. We obtained purified CD4^+^CD25^+^ T cells from the normal pregnant mice and the mice with *T. gondii* ESA-injection at G5, G10 and G15, respectively. The decreased suppressive ability of CD4^+^CD25^+^ T cells was observed in mice with the ESA-injection at G5 and G10. However, the inhibitory capacity of the CD4^+^CD25^+^ T cells was enhanced after the injection of *T. gondii* ESA at G15 (Figure 2D). Due to the capacity of CD4^+^CD25^+^ Treg cells controlling potentially detrimental IFN-γ reactions during pregnancy [28], we detected the serum level of IFN-γ after the injection of *T. gondii* ESA. We found that the serum level of IFN-γ was up to 448.3 pg/ml at G5 ip, suggesting that the activity of CD4^+^CD25^+^ Tregs on the suppression of IFN-γ production was impaired (Figure 2E). As expected, in all groups of mice, the serum IL-4 levels were not obviously affected (Figure 2F). Taken together, the results showed that the frequency and function of CD4^+^CD25^+^Foxp3^+^ T cells were diminished after the injection of *T. gondii* ESA at early and intermediate stages of pregnancy.

### Injection of *T. gondii* ESA at the Intermediate Stage of Pregnancy Decreases the Levels of Foxp3 mRNA and Protein at the Maternal-fetal Interface of Mice

**Figure 3:**
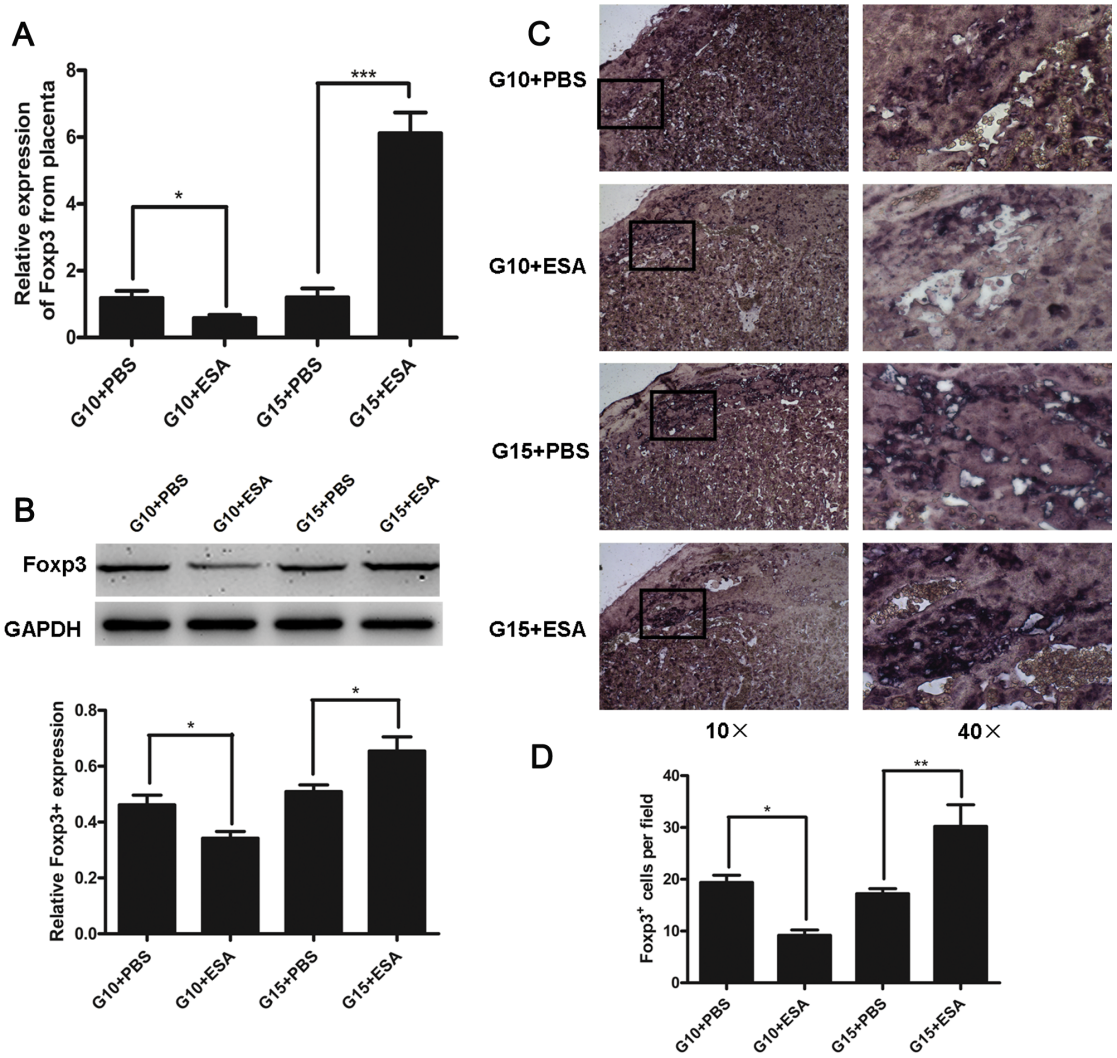
Foxp3 mRNA and protein levels at the maternal-fetal interface of mice with *T. gondii* ESA injection at G10 and G15. (A) Foxp3 expression levels in placentas from *T. gondii* ESA-injected and PBS-injected mice measured by real-time quantitative PCR. The data were normalized to individual β-actin mRNA expression and expressed as fold change relative to control mice. Data represent means ± SD from groups of seven mice assayed individually. (B)Top panel, Foxp3 protein was analyzed by Western blot after the injection at G10 or G15 as indicated. Bottom panel, densitometric analysis of Foxp3 expression was conducted by Western blot. One representative result of three independent experiments performed is shown. (C) The distribution of Foxp3^+^-cells in the placentas of *T. gondii* ESA-injected and PBS-injected mice as determined by immunohistochemical staining. (D) The average number of Foxp3^+^-cells per field. Data represent means ± SD from groups of four mice assayed individually. Statistical differences between groups are shown as follows: * *p*<0.05; **p<0.01; *** *p*<0.001.

A complex regulation of immune response at the maternal-fetal interface promotes tolerance of paternally derived antigens [29]. To determine if the reduction of CD4**^+^**CD25**^+^** Tregs also occurred at the maternal-fetal interface, we analyzed the expression levels of Foxp3 mRNA and protein in the placentas of mice with *T. gondii* ESA-injection at G10 and G15. The results showed that the expression levels of placental Foxp3 mRNA and protein were decreased at G10, but increased at G15, as compared with the control groups (Figure 3A and 3B). The distribution of Foxp3^+^ cells at the maternal-fetal interfaces was also observed by immunohistochemistry. As shown in Figure 3C and 3D, the placentas of mice with *T. gondii* ESA-injection at G10 exhibited decreased number of Foxp3^+^ cells, but that of mice with *T. gondii* ESA-injection at G15 presented increased number of Foxp3^+^ cells, as compared with the control groups. These data provided evidence that the injection with *T. gondii* ESA at G10 could lead to diminished number of Tregs, but the injection at G15 resulted in the increased number of Tregs at the maternal-fetal interface.

### The Capacity of CD4^+^CD25^+^ Tregs Favors the Maintenance of Pregnancy

**Figure 4:**
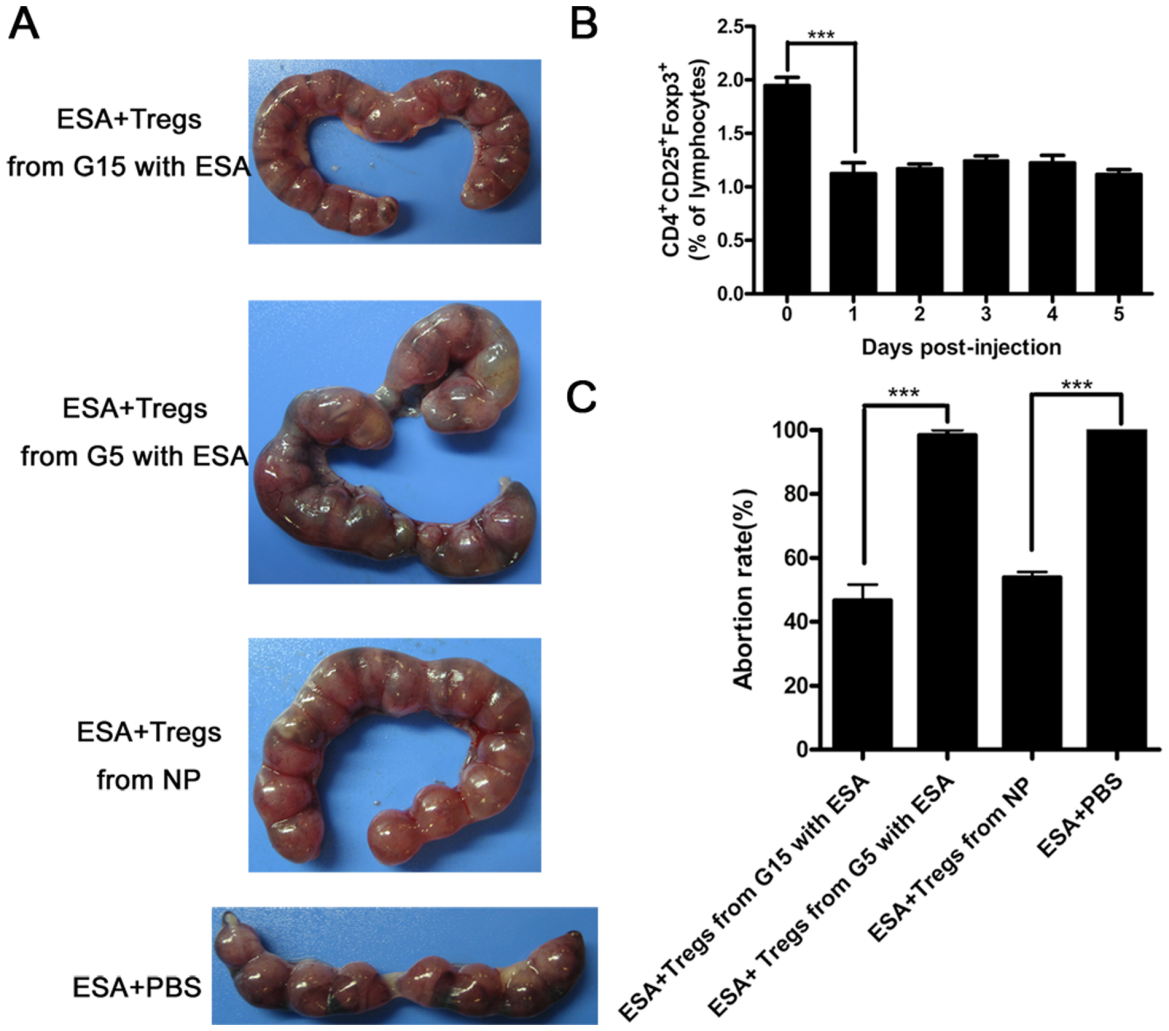
Reduced abortion rates in mice with *T. gondii* ESA injection at G5 by adoptively transfering of Tregs isolated from mice with *T. gondii* ESA injection at G15. (A) Representative pictures of uteri from the pregnant mice injected with *T. gondii* ESA at G5, which were transferred intravenously with 2×10^5^ freshly isolated CD4^+^CD25^+^T cells from the mice mentioned above at the first day post injection. The control mice were injected intravenously with 0.2 ml of sterile PBS at the same time. (B) The percentage of CD4^+^CD25^+^ Tregs was reduced at the first day post injection. * *p*<0.05 the comparison between 0 day and 1 day after post-injection with *T. gondii* ESA. (C)The abortion rates calculated as the ratio of abortion sites to the total number of implantation sites after the injection of *T. gondii* ESA. Data represent means ± SD from groups of four mice assayed individually. Statistical differences between groups are shown as follows: *** *p*<0.001.

To verify whether the diminished capacity of Tregs at G5 was causally associated with the fetal loss, we adoptively transferred CD4^+^CD25^+^T cells isolated from the spleens of normal pregnant mice, pregnant mice injected with *T. gondii* ESA at G5 or those at G15 into *T. gondii* ESA-injected pregnant mice at G5, respectively. First, we tested when the CD4^+^CD25^+^ Tregs decreased after the injection with *T. gondii* ESA. We found that the percentage of CD4^+^CD25^+^ Tregs significantly reduced to 1% at the first day post injection (1 dpi) (Figure 4B). Hence, we transferred Tregs to the abortion-prone mice at 1 dpi. We found that the adoptive transfering of CD4^+^CD25^+^ T cells from pregnant mice injected with *T. gondii* ESA at G5 failed to prevent the abortion. Interestingly, more than 50% abortion could be prevented by adoptive transfering of CD4^+^CD25^+^ T cells from pregnant mice injected with *T. gondii* ESA at G15 and normal pregnant mice (NP) (Figure 4A and 4C). Taking together, these data supported that the diminished capacity of CD4^+^CD25^+^Tregs caused by *T. gondii* ESA at early pregnancy directly led to the abortion of mice.

### *T. gondii* ESA Induces Apoptosis of Splenic CD4^+^CD25^+^ T Cells

**Figure 5:**
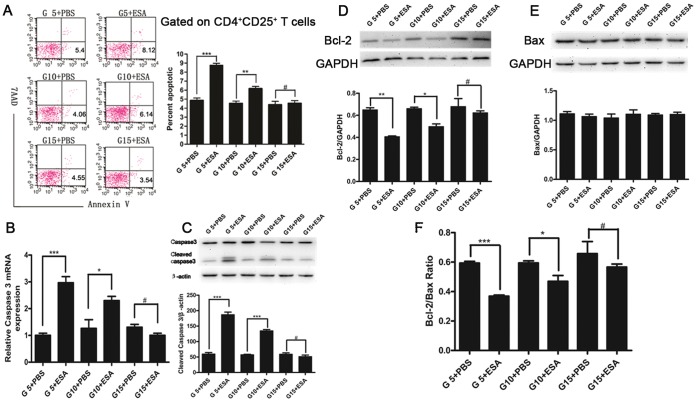
Apoptosis of CD4^+^CD25^+^ T cells in spleens of pregnant mice after *T. gondii* ESA injection at G5, G10 and G15. (A) A gate was set on CD4^+^CD25^+^ T cells, and the percentage of apoptotic CD4^+^CD25^+^ T cells was determined by Annexin V and 7AAD staining. (B) The expression of Caspase 3 from the purified CD4^+^CD25^+^ T cells at the level of mRNA. (C) The expression of Caspase 3 from the purified CD4^+^CD25^+^ T cells at the level of protein, which was quantified by densitometric analysis with Image J. (D) Top panel, Bcl-2 levels was analyzed by Western blot. Bottom panel, Bcl-2 levels were quantified by densitometric analysis with Image J. (E) Top panel, Bax levels was analyzed by Western blot. Bottom panel, Bax expression was quantified by densitometric analysis with Image J. (F) Bcl-2/Bax ratio. Statistical differences between groups are shown as follows: * *p*<0.05; ** *p*<0.01; *** *p*<0.001; ^#^ *p*>0.05.

To investigate whether apoptosis contributes to the reduction of CD4^+^CD25^+^ Tregs during *T. gondii* ESA injection at different stages of pregnancy, we performed flow cytometric analysis on splenocytes. As shown in Figure 5A, *T* *. gondii* ESA injection both at G5 ip and G10 ip, but not at G15 ip, could significantly induce apoptosis of CD4^+^CD25^+^ Tregs compared with the control. In line with the apoptosis, both mRNA and protein levels of activated Caspase-3 in CD4^+^CD25^+^ Tregs from mice with *T. gondii* ESA injection at G5 ip and G10 ip, but not at G15 ip, were increased significantly (Figure 5B and 5C). Also, the expression of Bcl-2 was decreased at G5 ip and G10 ip, but not at G15 ip (Figure 5D). However, the levels of Bax presented no obvious changes among the groups (Figure 5E). Therefore, *T. gondii* ESA injection at G5 ip and G10 ip led to decrease of Bcl-2/Bax ratio in splenic CD4^+^CD25^+^ T cells (Figure 5F).

### *T. gondii* ESA Induces Apoptosis at the Maternal-fetal Interface

**Figure 6:**
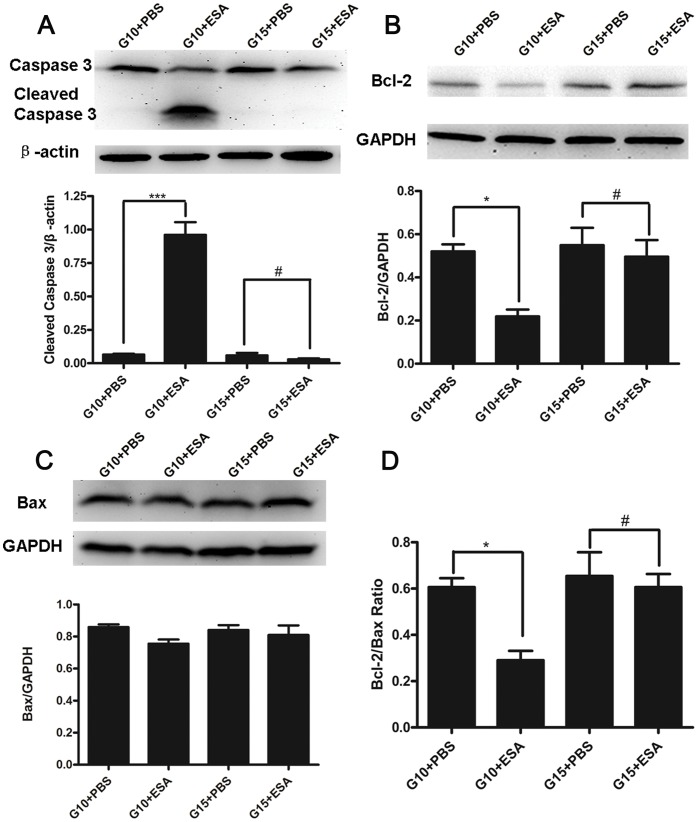
Apoptosis in local maternal-fetal tissues of pregnant mice with *T. gondii* ESA injection at G10. All data are presented as mean ± SE, and analyzed by one-way ANOVA. (A)Top panel, cleaved Caspase-3 expression was analyzed by Western blot after the injection with *T. gondii* ESA and PBS at G10 or G15. Bottom panel, cleaved Caspase-3 expression was quantified by densitometric analysis with Image J. (B) Top panel, Bcl-2 levels were analyzed by Western blot. Bottom panel, Bcl-2 expression was quantified by densitometric analysis with Image J. (C) Top panel, Bax levels were analyzed by Western blot. Bottom panel, Bax expression was quantified by densitometric analysis with Image J. (D) Bcl-2/Bax ratio. Statistical differences between groups are shown as follows: * *p*<0.05; *** *p*<0.001; ^#^ *p*>0.05.

To further investigate whether the apoptosis induced by *T*. *gondii* ESA at different stages of pregnancy occurred at local maternal-fetal tissues, the expressions of activated Caspase-3, Bcl-2, and Bax in placentas of variously treated animals were detected. The results showed that the cleaved Caspase-3 expressions were increased significantly in mice with *T. gondii* ESA injection at G10 ip, but not in mice with *T. gondii* ESA injection at G15 ip, when compared with the control groups, respectively (Figure 6A). Furthermore, the Bcl-2 expressions were decreased in the mice with *T. gondii* ESA injection at G10 ip, but not in mice with *T. gondii* ESA injection at G15 ip, when compared with the control groups, respectively (Figure 6B). However, as for the expressions of Bax, there was no statistically significant difference among the pregnant mice injected with *T. gondii* ESA or PBS at G10 ip and G15 ip (Figure 6C). Thus, the Bcl-2/Bax ratio was reduced in mice with *T. gondii* ESA injection at G10 ip compared with that in mice with *T. gondii* ESA injection at G15 ip (Figure 6D).

## Discussion

Toxoplasmosis is a serious disease in congenitally infected and immunocompromised individuals [30], [31]. Maternal infection of *T. gondii* at early and intermediate stages of pregnancy could result in severe congenital toxoplasmosis, while the infection that occurs at late pregnancy usually results in subclinical toxoplasmosis in their offspring [32]. Several lines of evidence suggest that early fetal resorption is due to other mechanisms rather than a direct effect of unterine *T,gondii* proliferation [16], [17]. Thus, in our study, *T. gondii* ESA was injected at different pregnant stages. We found that the injection of ESA at gestational days 5, 10, and 15 resulted in different outcomes, with abortion rates highest at the earlier time points of exposure to ESA (Figure 1). Our data supported the idea that early fetal resorption was due to the effect of *T. gondii* ESA.

During gestation, the maternal immune system normally tolerates the presence of paternal alloantigens. The mechanisms by which the maternal immune system tolerates semiallogeneic fetus without mounting immunological rejection are still limitedly understood. Normal pregnancy in human and mice is characterized by an increased number of Tregs in peripheral blood, decidua (uterus), lymph node and spleen. These increased number of Tregs have been shown to suppress alloreactive proliferation *in vitro* [33], [34], [35], [36], [37]. On the other hand, diminished number and function of Tregs will end in pregnancy failure [15]. We previously reported that *T. gondii* infection would result in fetal loss and decrease the number of splenic CD4^+^CD25^+^ regulatory T cells and placental Foxp3^+^ cells synchronously in the infected pregnant mice [17]. Given the effects of Tregs on pregnancy, we wondered whether Tregs were associated with different pregnancy outcomes. In this study, diminished number and function of Tregs were observed when the pregnant mice were injected with *T. gondii* ESA at early and intermediate stages of pregnancy. Interestingly, the number and function of Tregs were enhanced when the pregnant mice were injected with *T. gondii* ESA at late pregnancy. Based on our results that *T. gondii* ESA differently influence the number and suppressive function of Tregs at different stages of pregnancy, we presumed that the late pregnancy microenvironment-educated Tregs may resist *T. gondii* ESA stimuli. Further studies are required to demonstrate the functional differences of Tregs from different stages of pregnancy.

It was reported that the administration of anti-CD25 mAb at early pregnancy could induce implantation failure, while no effects were observed at late pregnancy [38]. However, anti-CD25 mAb may block not only Tregs, but also activated T lymphocytes [39]. In our study, to determine whether the diminished capacity of Tregs is causally associated with the fetal loss, we adoptively transferred CD4^+^CD25^+^ T cells isolated from the spleens of normal pregnant mice and pregnant mice injected with *T. gondii* ESA at G5 or at G15 and inject those cells into *T. gondii* ESA-injected pregnant mice at G5. Our data showed that the Tregs from the mice with *T. gondii* ESA injection at G15 ip reduced significantly the abortion rate, while the Tregs from the mice with *T. gondii* ESA injection at G5 ip failed to prevent the abortion. Apparently, these results demonstrated that the administration of *T. gondii* ESA did induce diminished capacity of CD4^+^CD25^+^ T cells at G5, and then resulted in the abortion. Although the adoptive transfering of Tregs failed to completely prevent abortion, it revealed that Tregs contributed partly, to the consequence. It is suggested that the different pregnancy outcomes of mice with the administration of *T. gondii* ESA at G5, G10 and G15 were due to the different effects of *T. gondii* ESA on the capacity of CD4^+^CD25^+^ T cells.

Our data indicated that the administration of *T. gondii* ESA at early pregnancy could lead to the decreased number of CD4^+^CD25^+^ T cells. Some studies demonstrated that CD4^+^CD25^+^ T cells can be regulated directly via triggering Toll-like receptor ligands [40], or indirectly via enhanced activation of APC or effector T cells [41]. In addition, enhanced apoptosis or a loss of function is also a causative event in regulating Treg cells [42], [43], [44]. Apoptosis of splenic CD4^+^ T cells during *Toxoplasma* infection has been observed by other researchers [45]. In a model of ocular toxoplasmosis, inflammatory cell apoptosis was implicated in disease pathogenesis [46]. T-cell apoptosis in the Peyer’s patches also accompanies intestinal necrosis during oral *T. gondii* infection [47]. However, the susceptibility of CD4^+^CD25^+^ Tregs to apoptosis is controversial. Human CD4^+^CD25^+^ T cells are apoptosis-prone because of lower expression of the antiapoptotic molecule Bcl-2 than conventional lymphocytes [48]. On the other hand, murine CD4^+^CD25^+^ T cells are resistant to apoptosis induced by Fas or dexamethasone [49], [50]. In our previous study, a small yet significantly higher proportion of splenic CD4^+^CD25^+^ T cells underwent apoptosis in pregnant mice infected with *T. gondii* than that in gestational age-matched controls [17]. In this study, after the administration of *T. gondii* ESA, the significant apoptosis of splenic CD4^+^CD25^+^ Tregs could also be observed at G5 ip and G10 ip compared with that of the control group. It is suggested that apoptosis should be contributive to the diminished number and function of Tregs after *T. gondii* ESA injection at early and intermediate stages of pregnancy.

Supposing that the changes in number and function of Tregs were related to apoptosis, we sought to further determine their possible mechanisms. Caspase-3 plays an irreplaceable role in apoptosis. Moreover, a recent study demonstrated that *T. gondii* might up-regulate the expressions of death receptor ligands; the subsequent binding of FasL to its receptor induces the trimerization of the receptor and formation of a death-inducing complex, which cause Caspase-8 activation, then cleaves various proteins and finally results in the activation of caspase cascades [51]. Besides, Bax and Bcl-2, the proteins of Bcl-2 family, serve as distinct regulators of apoptosis at its early stages. An interaction between Bcl-2 and Bax may be necessary for the apoptotic process to proceed [52], [53]. In this study, we confirmed that the administration of *T. gondii* ESA at G5 ip and G10 ip led to the up-regulation of cleaved Caspase-3 expression. Furthermore, we found the down- regulation of Bcl-2 at G5 ip and G10 ip and the up-regulation at G15 ip after *T. gondii* ESA administration, suggesting that the apoptosis induced by *T. gondii* ESA is through down-regulation of Bcl-2 expression. However, as for the expression of Bax, no significant differences were observed among mice with *T. gondii* ESA administration at G5 ip, G10 ip, G15 ip, although overexpression of Bax induced or enhanced spontaneous cell death without additional factors [54].

In conclusion, the present results demonstrate for the first time, to our knowledge, that *T. gondii* ESA injection at different gestational periods can differently influence CD4^+^CD25^+^ regulatory T cells and then lead to different pregnancy outcomes. The injection of *T. gondii* ESA at early and intermediate stages of pregnancy could result in the decreased frequency and impaired suppressive capacity of CD4^+^CD25^+^ regulatory T cells, while the administration that occurs at late pregnancy fails to impair those cells. In addition, the decreased frequency of CD4^+^CD25^+^ regulatory T cells is associated with the apoptosis caused by *T. gondii* ESA by down-regulating Bcl-2 expression and Bcl-2/Bax ratio. Our further study is focusing on the effects of *T.gondii* ESA on Tregs under estrogen/progestogen stress during pregnancy. In conclusion, the present study provides new insights into the mechanism that the abortion caused by *T. gondii* is more likely depending on the timing of pregnancy.
